# Situational analysis of general practitioners using a forecasting approach until 2025 and a multi-state Markov model: A retrospective longitudinal study

**DOI:** 10.51866/oa.379

**Published:** 2024-05-25

**Authors:** Azad Shokri, Fereshteh Farzianpour, Elmira Mirbahaeddin, Mahboubeh Bayat, Ali Akbari-Sari, Abbas Rahimi Foroushani, Iraj Harirchi, Somaieh Shokri

**Affiliations:** 1 BS, MSc, PhD, Social Determinants of Health Research Center,Research Institute for Health Development, Kurdistan University of Medical Sciences, Sanandaj, Iran. Email: azad_shokri@yahoo.com; 2 BS, MSc, PhD, Department of Health Management and Economics, School of Public Health, Tehran University of Medical Sciences, Tehran, Iran.; 3 BS, MSc, PhD, Telfer School of Management, University of Ottawa, 55 Laurier Ave E, Ottawa, Ontario K1N 6N5, Canada.; 4 BS, MSc, PhD, Center for Health Human Resources Research & Studies, Ministry of Health and Medical Education, Tehran, Iran.; 5 BS, MSc, PhD, Department of Health Management and Economics, School of Public Health, Tehran University of Medical Sciences, Tehran, Iran.; 6 BS, MSc, PhD, Department of Epidemiology and Biostatistics, School of Public Health, Tehran University of Medical Sciences, Tehran, Iran.; 7 MD, Department of Surgery, School of Medicine, Tehran University of Medical Sciences, Tehran, Iran.; 8 BS, MSc, Department of Physical Education and Sport Science, Mariwan Branch, Islamic Azad University, Mariwan, Iran.

**Keywords:** Unemployment, Immigration, General practitioners, Iran

## Abstract

**Introduction:**

Undesirable working conditions, insufficient professional development and other labour market pressures have significantly impacted the status of general practitioners (GPs). This study aimed to conduct a situational analysis of GPs in Iran using a forecasting approach until 2025.

**Methods:**

Data were collected concurrently through direct contact, data matching among databases and tracking among graduates from four clusters of medical science universities over the past decade. This retrospective longitudinal study determined the status of GPs over consecutive years. Multi-state Markov and binary logistic regression analyses were performed using R and Stata 14.

**Results:**

Of 430 graduates over the past decade, 94% were successfully identified. Only 20% of the graduates remained active as GPs. The greatest fluctuations in transfer occurred in the third year after graduation, with the remaining proportion of GPs dropping to less than 50%. The probability of remaining as GPs was 0.76 per year, while the highest transition was observed towards specialisation (0.12). Additionally, 2% of the GPs chose not to work, and less than 1% transitioned to a different specialty. Based on the transfer matrix for 2025, only 19% of the GPs were projected to remain, with the majority (59%) transitioning to specialisation.

**Conclusion:**

The transfer probability varies across different years, indicating higher flow rates among GPs. However, only a limited number of GPs are projected to remain until 2025. A comprehensive set of interventions should be considered, spanning the pre-medical stage, during education and after graduation, to mitigate the factors contributing to GPs leaving their profession.

## Introduction

General practitioners (GPs) are the first providers of healthcare for the community. They provide health services, implement and manage prevention programmes and respond to medical emergencies.^1,2^ Disagreeable job conditions and poor professional development opportunities in addition to other labour market pressures aflect their state and result in their displacement, leading to an outflow trend for these physicians.^3,4^ These flows significantly impact the projected human resource supply and the future human resource policies by which a population’s health needs are addressed. Despite the importance of this matter, collecting the required data to address GP flow has been a problematic issue for the Iranian health system. The system lacks the essential awareness of what is happening to doctors due to poor information resources.^5^ The lack of data on GP flow has become a concern across the country, particularly for policymakers interested in implementing an urban family physician programme for the first time in 2012 and 2006. The lack of reliable knowledge about the number of active GPs and the contradictory statistics in the early years of implementing a family physician programme in Iran have created concerns about its success.^2^ Two main areas of concern regarding GPs include their high unemployment rate, based on unrealistic statistics from the Iranian Medical Council data bank showing a record of 80,000 employed GPs in 2018, and unfeasible re-implementation of the family physician programme because of misleading statistics from other data banks estimating the number of active physicians to be less than the actual number required for the full implementation of the programme.^6,7^ The literature reveals that these inconsistencies in GP data are unavoidable due to significant difficulties in establishing an up-to-date record that effectively captures physicians actively practising medicine and their dynamic flow.^5^

In Iran, there appears to be a severe need for survey studies that inform the dynamics in the flow of GPs. The available data resources are scattered and not comprehensive; therefore, the present information on health human resource dynamics is limited and based only on estimates 8. Identifying different types of movements can help not only to clarify the subject but also to inform policies and determine the required measures. The present study aimed to analyse the status of GPs in Iran using a futuristic approach within 10 years until 2025.

## Methods

### Study design

This national longitudinal retrospective study was conducted among general medical graduates in Iran from 2008 to 2018. Link tracing was used to access data on each physician in the consecutive years after their graduation. According to the assumptions in this methodology, there is a network structure for data collection, and the sub-members provide the setting to identify the main members. In this study, the structure included three types of networks including classmates (friends), colleagues and relatives (secondary resources), who were identified through a matching process among the databases where they were registered as physicians, licensure data banks for colleagues or active databases of human resource offices including active physicians and other colleagues in the workplace. In case of relatives and nonprofessional acquaintances, a social media network was used.

In Iran, GPs are medical doctors who have completed a 7-year medical education and obtained a degree in general medicine. They have the option to pursue an additional 4 years of specialisation in specific medical fields at their university after general medicine. GPs serve as primary care physicians, providing comprehensive healthcare services that include diagnosing and treating various conditions, managing chronic diseases and offering preventive care.

### Sample size estimation

The sample size was calculated based on a confidence level of 95% and a margin of error of 0.5, considering the absence of similar studies conducted in Iran. Given that the general statistics of medical graduates were based on the national medical data bank including 120,000 records, the contribution of each item was 0.5 in the present study. The following formula was used:


$$\begin{array}{l} p=0.5\\ =\frac{1.96^{2}\times 0.5\times 0.5}{{\left(0.05\right)}^{2}}=384\text{n}=\frac{Z^{2}\times \text{p}\left(1-\text{p}\right)}{d^{2}} \end{array}$$

Considering the likely dropouts/loss of participants during sampling;, the loss rate was set to 0.15, and the number of the final sample was calculated as follows:


$$\text{n}*=\frac{384}{0.85}=452.$$

Given that the sample involved all graduates during the last decade (29,008 graduates), the sample was defined for a limited population:


$$\text{n**=}\frac{\text{n*}}{1+\frac{\text{n*}}{\text{N}}}=\frac{452}{1+\frac{452}{8,523}}=430.$$

A list of the total alumni at medical science universities during the past decade was prepared, and three cohorts with at least 2 years of difference were selected to analyse their activities (cohorts 2008, 2010 and 2012). The sample sizes for each cohort were determined proportionately to the size of each cohort. The medical science universities were organised into four clusters based on a ranking list published by the Ministry of Health and Medical Education (MOHME) in the RAD Project in 2018 to cover all graduates in the country. The university ranking system or RAD, initiated by the Deputy of Education at the MOHME, serves the purpose of identifying weaknesses, promoting cohesive and coordinated efforts between the Ministry of Health and universities to address educational challenges, guiding universities towards overarching goals in assessing higher education in the country and fostering a spirit of collaboration in a healthy and competitive environment to expedite the progress of medical universities nationwide. Clusters one, two, three and four corresponded to the universities with ranks ≤3, 4–13, 14–37 and ≥38, respectively.

### Data collection

**Figure 1:**
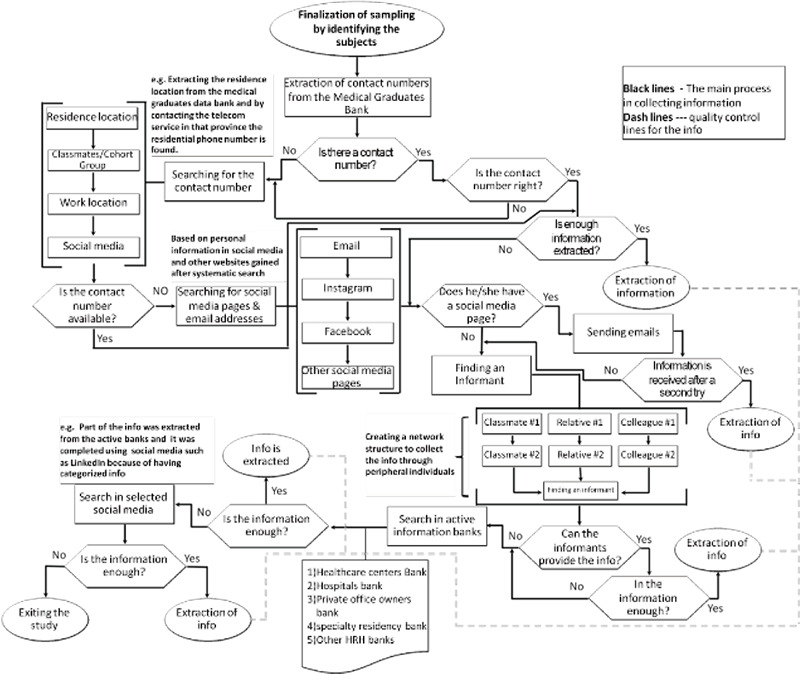
Datacollectionprocessamonggeneral practitioner graduates.

Figure 1 illustrates the data extraction process. The main focus of this study was on ensuring direct contact with participants and gathering information firsthand. Most efforts were made to ensure direct contact with participants. When physicians were neither available on social media, responding to emails nor willing to have a telephone conversation, other routes such as secondary resources were used. With this approach, a network structure was created for collecting information through secondary resources. From the active physicians’ data bank, the researchers accessed the phone numbers of classmates in the same cohort and other physicians’ workplace contact number in recent years until data were complete. Finally, for physicians whose data could not be collected from either of the resources, a matching model was adopted by which physicians were matched with the present active banks, and their working locations were identified in recent years. Social media platforms such as Facebook and year-to-year information from reliable scientific websites or academic/university webpages (e.g. researchgate.com) were used to complete data on some physicians.

The extracted results were measured using at least one of the other methods to increase data confidence. The confidence path is illustrated with dash lines in Figure 1.

For data collection, a checklist was employed. This checklist encompassed various individual characteristics such as name, sex, age, year of graduation and university (Supplementary 1).

### Statistical analysis

Data on each state were extracted per year for each period. Thereafter, the existing status matrix was calculated using a multi-state Markov model. The year participants were sent for service commitment was considered the starting point. Therefore, all participants passed a year after graduation within the first year of the study.

The transition matrix from the current state to the new state for each mode was predicted for the year 2025. Approximately 70% of the estimates were used to measure the remaining 30% to verify the validity of the predictions. A P-value of <0.05 was used to evaluate the validity of the model comparing the predicted probabilities with the collected data. The correlations of the estimated results for 3 years and the actual results were calculated. The correlation coefficient of the two matrices was 0.95, showing a substantially high correlation. Finally, based on the obtained data about each participant including their demographic characteristics, university at the time of graduation, year of graduation and other characteristics, a logistic regression analysis was conducted to examine the relationship between these variables and the status of participants years after graduation. The analyses were performed using the statistical software R.

### Ethical approval and consent to participate

All procedures were approved by the Ethics Committee of the Tehran University of Medical Sciences under ethics code number IR.TUMS. VCR.REC.1399.1045. Written/oral informed consent was obtained from all participants.

## Results

### Demographic characteristics

Out of 430 graduates, 397 (94%) were included in this study. Eight graduates were excluded because of their foreign citizenship (four GPs), not having a medical council number, non-inclusion in the medical system bank (three GPs) and lack of participation (one GP). Table 1 shows that most participants (66%) were aged 30–35 years, and 59% were women. The majority (37%) graduated in 2012 and attended secondary cluster universities (36%). More than half of the participants pursued residency or were in service as medical specialists. Approximately 9% had migrated to other countries (54% to the USA, 17% to Germany, 12% to Australia, 5% to Canada and 10% to other countries including Canada, South Africa, the UAE, English-speaking countries, Italy and Turkey), and 8% were not practising due to unemployment (2.5%), unwillingness to practise (1.8%) or preparation for the residency entrance examination (4%). In total, only 20% of the participants were still active in the field of general medicine (Table 1).

**Table 1:** Demographic characteristics and current status of the physicians.

	Variable	n	%
Sex	Male Female	163 234	41 59
Age, year	<35 35-40 >40	263 123 11	66 31 3
Year of graduation	2012 2010 2008	145 130 122	37 33 31
University category	First Second Third Fourth	51 142 116 88	13 36 29 22
Current status	Specialist General practitioner Immigration Unemployment Reluctance to work Preparation for the residency entrance examination Health-related employment^*^ Departure from the profession^**^	219 79 37 10 7 16 25 4	55 20 9 3 2 4 6 1
**Total**		**397**	**100**

* Graduates who work in non-clinical settings including headquarters or physicians who work in clinical fields that are unrelated to general medical tasks, including drug addiction, skin and hair or medical equipment fields

** Physicians who work outside the healthcare field and in other areas such as businesses (e.g. construction-related businesses)

### Probability of transition to different states

**Figure 2:**
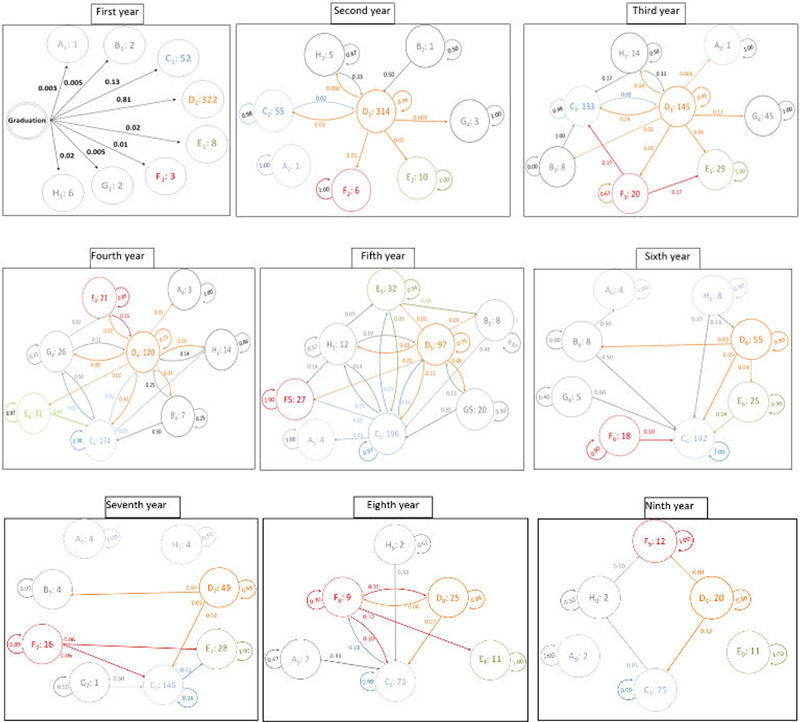
Probability of transfer among medical graduates years after graduation. A: Departure from the profession (non-health-related employment); B: Unemployment; C: Medical specialist; D: General practitioner; E: Immigration; F: Health-related employment; G: Preparation for the residency entrance examination; H: Reluctance to work. A1: 1 indicates that in the first year, there was one individual who left the profession (non-health-related employment); E5: 32 indicates that in the fifth year, there were 32 individuals who had immigrated; D7 : 49 indicates that in the seventh year, there were 49 individuals who practised as general practitioners; other numbers in various years are likewise interpreted based on these instances.

Figure 2 shows the probability of any state transition among the participants years after graduation by year. Fluctuations started from the first year, and in that year, different transition states were noted: departure from the profession (non-health-related employment), unemployment, medical specialist, GP, immigration, health-related employment, preparation for the residency entrance examination and reluctance to work. In the first year after graduation, the probability of transition to general medicine was 0.8, indicating that 80% of all participants entered the general medical practice during the immediate year after graduation. The probability of transition to specialisation was 0.13. The greatest fluctuations occurred in the third year of graduation. The overall remaining percentage of GPs reached the lowest level (less than 0.5 or 50%). Consequently, of 314 GPs in the second year, only 145 remained in general practice. Approximately 13% refrained from practice to prepare for the residency entrance examination, and 100% of the unemployed GPs (from the previous cohorts) entered residency. After the third year, there was a steady decrease in the fluctuations of the GP states, while the retention rate in general practice increased. For instance, the retention rate in general practice was 45% in the third year, which increased to 73% in the fourth year and 89% in the sixth year. Conversely, the rate of attrition from general practice followed a decreasing trend.

**Figure 3:**
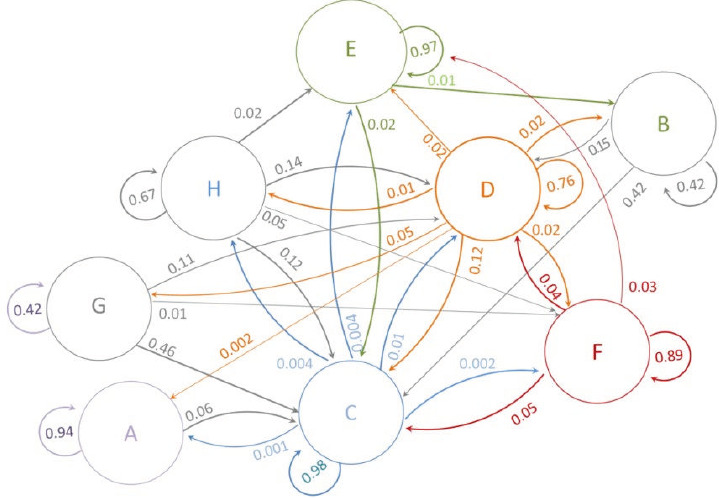
Probability of transition among medical graduates 10 years after graduation. A: Departure from the profession (non-health-related employment); B: Unemployment; C: Medical specialist; D: General practitioner; E: Immigration; F: Health-related employment; G: Preparation for the residency entrance examination; H: Reluctance to work.

Figure 3 shows the transition probability years after graduation. During this period, the average rate of remaining in general practice was 0.76 per year. The highest transition probability was noted with starting a specialty (0.12) and the lowest with preparing for the residency entrance examination (0.05). The highest transition probability was observed from the preparation for the residency entrance examination and unemployment to specialisation (0.46 and 0.42, respectively). Approximately 2% of the participants who were reluctant to work and less than 1% of the participants who started a specialty transitioned to immigration.

**Figure 4:**
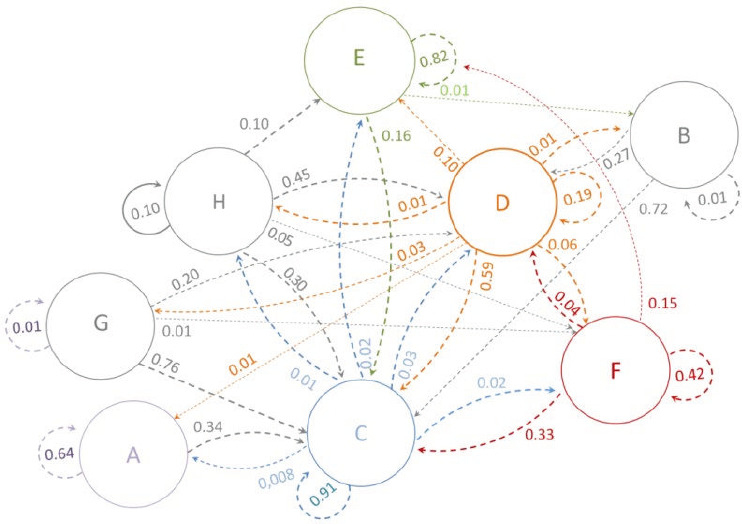
Predicted probability of transition in 2025. A: Departure from the profession (nonhealth-related employment); B: Unemployment; C: Medical specialist; D: General practitioner; E: Immigration; F: Health-related employment; G: Preparation for the residency entrance examination; H: Reluctance to work.

Figure 4 shows the transition matrix from the existing state to a new state in 2025. According to the existing trends, only 19% were predicted to remain in their state over the next 8 years, while 59% were anticipated to transition to the specialisation state. Approximately 10% were expected to migrate to other countries and 6% to engage in businesses other than practising medicine. Conversely, the highest probabilities of transition to specialisation were noted among the participants who were unemployed and those preparing for the residency entrance examination (0.76 and 0.72, respectively). Approximately 10% of the participants who were reluctant to work and 2% of those who were specialists were predicted to emigrate in the next 8 years, while 16% of those who had already migrated were anticipated to return and re-enter specialisation.

### Predictors of transition to different states

As shown in Table 2, sex and year of graduation were not significant predictors of transition to different states (P<0.05). Conversely, transition was significantly affected by age, status of service commitment and university cluster (P<0.05). For example, the participants who did not complete the service commitment had a 64% lower probability of transitioning to different states (odds ratio [OR] =0.36, confidence interval [CI]=0.13–0.95, P=0.04). The probability of transitioning to different states decreased by 7% per year of age increase (0R=0.93, CI=0.86–1.00, P<0.05). Moreover, a lower level of university education was associated with a higher probability of transitioning. The participants who graduated from fourth-class universities had a 3.5-fold higher probability of transitioning than those who graduated in 2012 (CI=1.15–10.84, P=0.04). The predictors of each state are detailed in Supplementaries 2–8.

**Table 2:** Impact of the demographic and educational characteristics on the status after graduation.

		Odds ratio	95% Confidence interval		P
			Lower limit	Upper limit	
Sex	Male	1.00			
	Female	1.11	0.63	1.96	0.72
Age		0.93	0.86	1.00	*0.05*
Completion of service commitment^*^	Yes	1.00			
	No	0.36	0.13	0.95	*0.04*
	First	1.00			
University category	Second	0.99	0.43	2.29	0.99
	Third	1.67	0.67	4.15	0.27
	Fourth	3.53	1.15	10.84	0.03
	2012	1.00			
Year of graduation	2010	1.15	0.59	2.23	0.68
	2008	1.54	0.73	3.24	0.25

* Service commitment: The compulsory services law for physicians and allied workers mandates all newly graduated physicians to offer their services in an underserved area before they can proceed with further education or commence their private practice.

## Discussion

The study showed that only 20% of the graduates were still active in the field of general medicine, which was anticipated to decline and reach its lowest level in 2025. The majority of the graduates experienced a variety of different states years following their graduation and chose their state according to their circumstances. The results also showed that the highest fluctuation occurred in the third year. One likely hypothesis is that the third year is when physicians’ obligation for the service commitment is finished. According to the law of service commitment in Iran, graduates from all medical schools are obliged to have 2 years of full-time service.^9^ Thus, the level of fluctuation sharply increased by the end of the legal commitment in this study. Following this period, the probability of the GPs remaining in general practice and moving to another state was 0.76 and 0.23 per year, respectively. In 2025, only 20% of the GPs were expected to remain in general practice. Of the total of 79 GPs in 2018, fewer than 16 GPs were estimated to remain in general practice in 2025, indicating a significant decline in GPs’ interest in general medicine in the years following graduation. As expected, after 20 years of graduation, GPs completely leave the field. Studies focusing on physician retention in underserved areas showed that service commitment policies are effective in the short term and depend only on the years of the retention policy.^10,11^

The present results exhibited that most transitions involved specialisation years after graduation. Several studies have been conducted about physicians’ eagerness to pursue a specialty in both high- and low-income countries. Approximately 97.6% of students in Greece, 95.3% in India, 89% in Sudan and 89.5% in Africa have reported a high tendency towards residency and a career in specialised fields.^12-15^ Studies from more developed countries have observed a varied pattern. In Canada, a tendency towards specialisation has increased annually (e.g. from 38% to 43% in 2003) 16. However, this trend differs from that in the UK, the USA and most European countries.^17,18^

Regarding immigration, the current study showed a steady and growing trend of outmigration to other countries in all periods. From 1991 to 2004 in Turkey, the average out-migration rate among physicians ranged from 3.2% to 3.8%. Migration has been one of the key elements of Turkish–German relations as noted in the medical history of both countries.^19^ In South Africa, 84.8% of students have been reported to have an inclination to immigrate. Conversely, the immigration rate is 50% for Polish students^20^, 60% for Lithuanian doctors^21^ and 45% for Czech doctors.^22^ In Ireland, the immigration rate among GPs slowly increased from 16.9% in 2014 to 19.2% in 2017. Proximity to developed European countries is one of the reported reasons for this difference.^23^ In the UK, the immigration rate reached 35% and increased by 0.25% in 2014.^24^

Concerning job departure or unemployment, this study distinguished participants who were unemployed from those who were reluctant to work in the medical field and those who were preparing for the residency entrance examination. The statistics among these participants were classified individually. In this study, the probability of transition from general medicine to unemployment was 2% per year; from general medicine to unwillingness to work, 1%; and from general medicine to preparation for the residency entrance examination, 5%. In contrast, a study conducted in Iran showed a 9.4% unemployment rate. Rad et al. showed an unemployment rate of 7.8% for physicians^25^, while the national statistics report of Iran in 2011 declared a rate of 12.3%. A study conducted in the Kerman University of Medical Sciences in Iran showed that 7.6% of the total graduates were unemployed 3 years after graduation.^26^ These previous findings are consistent with the present findings on the number of physicians without any occupation owing to unemployment, unwillingness to work or preparation for the residency entrance examination.

Notably, the present study showed that in the third year after graduation, which concurs with the end of the service commitment, the highest transition rates were noted with preparation for the residency entrance examination. In the following year, the majority of the GPs transitioned to specialisation. Therefore, most GPs who entered the specialty field had 1 year of unemployment and then sat for the residency entrance examination. Previous research has indicated that exposing students to specialised areas early during clinical training can stimulate their academic interest.^27^ Ultimately, leaving general practice may be aimed at employment in other areas of health such as government health headquarters or other clinical settings including drug addiction and substance abuse, skin, acupuncture or research and education centres. Otherwise, physicians engage in completely unrelated fields, such as managing industrial plants or educational institutions. The main reasons doctors consider leaving the UK are lifestyle choices and working conditions. Lifestyle choices include seeking a better quality of life, personal growth, relief work abroad and travel. Working conditions include poor conditions, low pay and long working hours. Some doctors also mention achieving positive work-related goals as their reason for leaving. Those with work-related reasons aim to enhance their medical experience through international opportunities.^28^

Limited studies have evaluated the present topic. In a study conducted in Malawi in 2014, 6.9% of graduates were reported to work in other health-related settings, including research centres.^29^ In a study among female medical students in England, 1% reported wanting to work outside healthcare.^30^ Many graduates appeared to enter other businesses because of the inability to achieve satisfactory conditions by practising medicine in terms of financial attainment and working conditions.

Generally, the fluctuation rates are significant among general medical graduates in Iran. With the fluctuations in the next 8 years until 2025, only a limited number of GPs are anticipated to remain in general practice. These results are contradictory to the policies adopted for the implementation of family medicine in recent years and the statistics on active GPs of the national medical council. The lack of awareness among policymakers and authorities about these fluctuations can challenge their decisions in the future years with respect to training GPs and providing workforce for implementing various measures such as family medicine programmes.

## Conclusion

Despite the study’s limitations, such as the small sample size of physicians and the potential challenges in generalising the findings to the entire physician population, the study represents the first study to retrospectively examine the status of physicians in Iran. The results indicate significant fluctuations in the percentage of general medical graduates in Iran. A diverse range of interventions is recommended to address the factors contributing to the departure of GPs. These interventions encompass reforms before pursuing a medical degree, reforms during medical education at universities and post-entry reforms within the medical field. Such reforms may include initiatives to inform prospective students about working conditions, opportunities for local students to study and train in local medical universities and long-term reforms to improve societal attitudes towards the medical profession and enhance the functioning of family referral and medical systems.

## Data sharing statement

Data sharing is not applicable, as no datasets were generated or analysed during the study.

**How does this paper make a difference in general practice?**

- This paper presents a situational analysis of general practitioners (GPs) in Iran and forecasts their status until 2025.
- It highlights the challenges faced by GPs, such as poor working conditions and limited professional development opportunities, leading to a high outflow of physicians from the profession.
- The study analyses data collected from graduates over a decade, revealing fluctuations in the number of active GPs and their transition patterns towards specialization.
- The findings emphasize the need for comprehensive interventions to retain GPs, including improvements in working conditions, increased professional development, and support throughout their education and career.
- This research contributes to the understanding of GP dynamics and provides valuable insights for policymakers to develop strategies ensuring a sustainable and eflective GP workforce.
